# Efficacy of psychological interventions for irritable bowel syndrome: A protocol for systematic review and meta-analysis

**DOI:** 10.1097/MD.0000000000029033

**Published:** 2022-03-11

**Authors:** Fei Yao, Xutao Wu, Huacheng Zhao, Chun Gan

**Affiliations:** Department of Gastroenterology, the Second Affiliated Hospital of Jiangxi University of Traditional Chinese Medicine, Jiangxi, China.

**Keywords:** efficacy, irritable bowel syndrome, meta-analysis, psychological interventions

## Abstract

**Background:**

Guidelines for the management of irritable bowel syndrome (IBS) recommend that psychological therapies should be considered, but their relative efficacy is unknown. We performed a protocol for systematic review and meta-analysis to try to resolve this uncertainty.

**Methods:**

Two individual researchers conducted the platform searches on Ovid Medline In-Process & Other Non-Indexed Citations, Ovid MEDLINE, Ovid EMBASE, Ovid PsycINFO, Ovid Cochrane Central Register of Controlled Trials, Ovid Cochrane Database of Systematic Reviews, and Scopus from inception to February 2022. Systematic review and meta-analysis of the data will be performed in STATA13.0 software according to the preferred reporting items for systematic reviews and meta-analyses protocols guidelines. Two authors independently performed the literature searching, data extraction, and quality evaluation. Risk of bias was assessed using the Cochrane Risk of Bias Tool for randomized controlled trials.

**Results:**

A synthesis of current evidence of psychological interventions for IBS will be provided in this study.

**Conclusion:**

This result will provide a comprehensive analysis and synthesis to inform practitioners and policy makers about the effectiveness of psychological interventions for patients with IBS.

## 1 Introduction

Irritable bowel syndrome (IBS), a chronic gastrointestinal condition, affects as many as 10% of people.^[1]^ Historically, IBS has been defined as a functional bowel disorder, but more recently it has been recognized as a disorder of gut–brain interaction.^[2,3]^ IBS is characterized by abdominal pain in association with a change in stool frequency, and/or form.^[4]^ The pathophysiology is multifactorial, and includes disturbed gastrointestinal motility, visceral hypersensitivity, and altered central nervous system processing^[5,6]^; however, the mechanisms by which these processes interact are poorly understood. Thus, IBS is difficult to manage clinically and, as a result, this chronic episodic condition impacts considerably on social functioning and quality of life.

Recently, acknowledgment of the role of stress and psychosocial factors in some cases has led to the examination of psychological therapies targeting these factors in the treatment of IBS.^[7,8]^ In the past few decades, research has uncovered an extensive bidirectional communication network between the brain and the gut termed the brain–gut axis. This provides a pathophysiologic basis for the potential therapeutic effects of psychological therapies on gut function. This has been further supported by several, small, randomized controlled trials (RCTs) demonstrating the preliminary efficacy of psychological therapies on IBS symptoms.

Hypnotherapy has been suggested to treat abdominal pain, improve quality of life, and reduce anxiety and depression in IBS without any side effects.^[9]^ These effects persisted for several years, although definitive conclusions will require larger, higher quality studies. Cognitive behavioral therapies and mind-body therapies have also been studied in IBS with some studies showing preliminary efficacy.^[10,11]^ Psychological therapies are potentially efficacious in treating IBS symptoms in many patients,^[12]^ and unlike many pharmaceutical treatments, they have minimal side effects and can be cost-effective. To examine if psychological therapies merit incorporation in the clinical treatment of IBS, we conducted this protocol for systematic review and meta-analysis of published RCTs.

## 2 Methods

### 2.1 Protocol register

This protocol of systematic review and meta-analysis has been drafted under the guidance of the preferred reporting items for systematic reviews and meta-analyses protocols.^[13]^ It has been registered on open science framework (Registration number: 10.17605/OSF.IO/PY8TX). Ethical approval is not required for this study since it relies on secondary data.

### 2.2 Eligibility criteria

We included RCTs that enrolled patients of unspecified gender and aged at least 18 years. Subjects of the included trials were diagnosed with IBS based on one of the following criteria: Latimer criteria, Manning criteria, Kruis criteria, Rome I criteria, Rome II criteria, Rome III criteria, or clinician defined diagnosis. We included trials that evaluated the efficacy of psychological interventions, including cognitive–behavioral therapies, mind–body therapies, and other psychological interventions, compared with no intervention, waiting list, placebo, diet, herbal treatment, or symptomatic management. The primary outcomes were the composite IBS symptoms severity scales and quality of life. Other outcomes were diarrhea, constipation, and abdominal pain.

Nonrandomized comparative studies and single arm studies were not included. We excluded trials that evaluated hypnotherapy because multiple systematic reviews have already summarized this evidence.

### 2.3 Search methods

A comprehensive search of several databases from 1966 to February, 2022 was conducted. The databases included Ovid Medline In-Process & Other Non-Indexed Citations, Ovid MEDLINE, Ovid EMBASE, Ovid PsycINFO, Ovid Cochrane Central Register of Controlled Trials, Ovid Cochrane Database of Systematic Reviews, and Scopus. Two authors will independently draft and carry out the search strategy. In addition, we manually retrieve other resources, including the reference lists of identified publications, conference articles, and gray literature. The key terms used for the search were “psychological,” “irritable bowel syndrome,” and “randomized controlled trial.”

**Figure 1:**
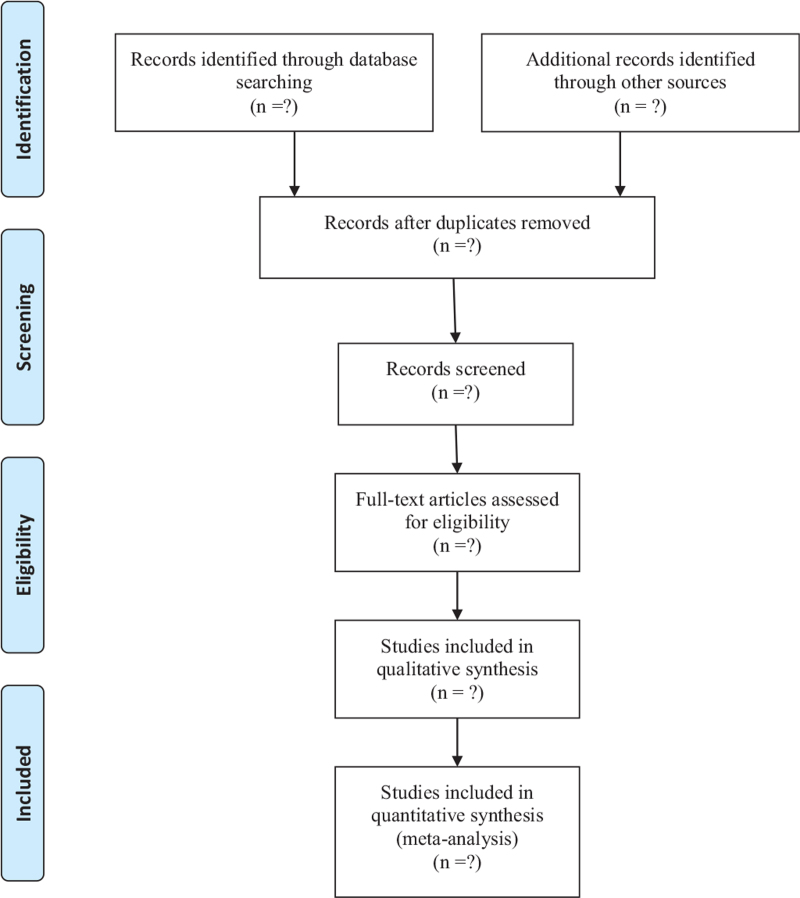
Flow diagram of study selection.

The retrieval process is presented in Fig. 1.

### 2.4 Data extraction

The following data were extracted for each article: bibliographical data, including authors and year of publication; clinical trial features such as sample size, study flow, recruitment method, criteria for inclusion and exclusion, primary measures, time and point of assessments, and duration of the intervention; participant characteristics such as age, sex, and so on; patient background, including country and race; and study drop-out rate and handling of missing data. Data extraction was performed by 2 independent investigators according to a predesigned review form. Disagreements were resolved through discussion among all authors.

### 2.5 Assessment of the risk of bias

Two reviewers evaluated the methodological quality of the included trials according to the Cochrane Risk of Bias tool.^[14]^ We evaluated the adequacy of randomization, allocation concealment, blinding (patients, providers, data collectors, and outcome assessors), baseline imbalance, and extent of loss to follow-up. The bias of the study will be rated on 3 levels: “low,” “high,” and “ambiguous.” We also extracted the funding source.

### 2.6 Statistical analysis

Because the outcomes of interest were evaluated in the included trials using different scales, we estimated the standardized difference in means (SMD) to measure the difference between the intervention and control groups. SMD calculation involves standardizing the effect and expressing it in standard deviation units, to allow pooling it across trials. For each trial, we calculated the change in the studied scales before and after the intervention and compared it with the change in the control group. Then DerSimonian and Laird random-effects model was used to pool SMD across trials.^[15]^ Inconsistency across the trials was assessed using the *I*^2^ static and Cochran *Q* test. *I*^2^ value >50% was considered indicative of substantial heterogeneity that is due to real differences in protocols, trial populations, interventions, and/or outcomes. Also, Cochran *Q* test *P* value <.05 indicates that the heterogeneity is beyond chance or random error. We planned to conduct formal tests to assess potential publication bias using visual inspection of funnel plots and Egger regression asymmetry tests. Two researchers respectively entered the data into the STATA13.0 software (Stata Corp, College Station, TX).

## 3 Discussion

Psychological interventions have been designed and implemented effectively in a wide range of medical conditions. The subspecialty area of clinical health psychology aims specifically to identify and target stress-related and psychological factors that may contribute to the impact or expression of medical problems. Over the past several decades, health psychology and gastroenterology have become increasingly aligned, with a large body of research to support the effectiveness of psychological interventions for a range of gastrointestinal disorders.

Given that IBS has been recognized as a disorder of gut–brain interaction,^[16,17]^ it is becoming increasingly understood how psychological comorbidity may have an impact on gastrointestinal function and vice versa, although cause-effect mechanisms remain unclear. Gastrointestinal-focused psychological and behavioral therapies can target brain–gut dysregulation and are beneficial in some patients.^[18]^ Although these treatments have effects within the central nervous system, they also have peripheral effects on pain perception, visceral hypersensitivity, and gastrointestinal motility. This article evaluates the evidence for psychological therapies in IBS treatment, more high quality RCTs will be required to confirm the conclusion.

## Author contributions

**Conceptualization:** Xutao Wu.

**Data curation:** Xutao Wu, Huacheng Zhao.

**Funding acquisition:** Chun Gan.

**Investigation:** Huacheng Zhao.

**Methodology:** Huacheng Zhao.

**Writing – original draft:** Fei Yao.

**Writing – review & editing:** Chun Gan.
